# *LRP4*-Related Congenital Myasthenic Syndrome: Clinical, Pathophysiological, and Therapeutic Aspects

**DOI:** 10.3390/muscles5030046

**Published:** 2026-06-24

**Authors:** Felipe Yuji Koyama Azeka, Paulo de Lima Serrano, Daniel Delgado Seneor, Sophia Luiz Calegaretti, Mateus Medeiros Pinheiro, Marcos Vinícius Martins, Leonardo Mobiglia Guilherme, João Paulo Barile, Patrícia Marques Mendes, Lucas Henrique de Moura Rogério Garcia, Maria Júlia Tabosa de Carvalho Galvão, Sofia Mônaco Gama, Renan Brandão Rambaldi Cavalheiro, Igor Braga Farias, Roberta Ismael Lacerda Machado, Wladimir Bocca Vieira De Rezende Pinto, Acary Souza Bulle Oliveira, Paulo Sgobbi

**Affiliations:** 1 Division of Neuromuscular Diseases, Department of Neurology and Neurosurgery, Federal University of São Paulo (UNIFESP), São Paulo 04039-060, Brazil; felipe.kazeka@einstein.edu.br (F.Y.K.A.); patricia.marques.11@hotmail.com (P.M.M.); pvsgobbi@gmail.com (P.S.); 2 Einstein Medical School, Faculdade Israelita de Ciências da Saúde Albert Einstein (FICSAE), São Paulo 05653-120, Brazil; 3 Santa Casa de São Paulo School of Medical Sciences (FCMSCSP), São Paulo 01221-020, Brazil; 4 Ingá University Center (UNINGÁ), Maringá 87035-510, Brazil

**Keywords:** LRP4, congenital myasthenic syndrome, neuromuscular disorders, neuromuscular junction, Cenani-Lenz syndactyly syndrome, sclerosteosis

## Abstract

Congenital Myasthenic Syndrome represents a complex and heterogeneous group of inherited neuromuscular disorders, which result from variants in genes involved in different pathophysiological mechanisms related to the neuromuscular junction. LRP4 (Low-density lipoprotein receptor-related protein 4) represents one of the most important proteins involved in this complex signaling pathway, acting in a complex with agrin and Muscle Skeletal Receptor Tyrosine Kinase (MuSK) proteins. LRP4 became known to most neurologists due to the description of anti-LRP4 antibody-related Myasthenia Gravis. There are, however, different neurological and neuromuscular disorders that result from pathogenic variants in *LRP4* gene, especially a rare and potentially treatable Congenital Myasthenic Syndrome. The authors performed a detailed narrative review, including descriptions of the main pathophysiological, clinical, and therapeutic aspects of *LRP4*-related Congenital Myasthenic Syndromes.

## 1. Introduction

Neuromuscular junction (NMJ) formation and maintenance require a complex signaling pathway involving motor neurons and skeletal muscle fibers [1]. Understanding the genes involved in the main functional mechanisms of the neuromuscular synapse is important not only to discover its normal organization but also to provide insight into neuromuscular disorders termed Congenital Myasthenic Syndromes (CMS) [2]. A total of 40 genes have been identified as associated with CMS (Table 1) [3]. The genes can be classified into three categories: presynaptic, synaptic, and postsynaptic CMS. Ohno and collaborators classified the pathogenic variants of CMS into 13 subtypes according to clinical, pathomechanical, and therapeutic features.

The prevalence of CMS per million in the overall population is 2.2 and 9.7 in individuals under 18 years of age. However, the prevalence varies considerably between geographical locations. In the United Kingdom, a study of 123 CMS patients identified that the prevalence of CMS in individuals under 18 years old differs according to geographic location, ranging from 2.8 to 14.8 per million, with an average of 9.2 per million [4]. The prevalence of CMS per million in the total population in Slovenia, Spain, Austria, and Belgium is 22.2 (8/360,000), 1.8 (64/35,500,000), 3.1 (28/9,000,000), and 3.2 (37/11,900,000), respectively [5,6,7,8]. In the Southern Brazilian state of Parana, the prevalence in 2010 was 1.8 per million (1.8/1,000,000), which reinforces the lack of recent epidemiological research in the country [9]. Notably, there is an underestimated prevalence due to undiagnosed CMS patients, according to an analysis of all reports [5].

**Table 1:** Summary of CMS phenotypes and the involved genes. Legends: AChE: acetylcholinesterase; AChR: acetylcholine receptor; CMS: Congenital Myasthenic Syndrome; FCCMS: fast-channel congenital myasthenic syndrome; LEMS: Lambert–Eaton myasthenia syndrome; SCCMS: slow-channel congenital myasthenic syndrome [3,10,11].

Phenotype	Gene
**Presynaptic**	
Defects in acetylcholine synthesis and recycling	*CHAT*, *SLC18A3*, *SLC5A7*, *PREPL*
LEMS-like CMS	*SYT2*, *SNAP25*, *MUNC13-1*, *VAMP1*, *RPH3A*, *LAMA5*
Defective nerve terminal formation	*MYO9*, *SLC25A1*, *TEFM*
Sodium channel CMS	*SCN4A*
**Synaptic**	
Defects in AChE	*COLQ*
Defects in synaptic basal lamina and matrix	*LAMB2*, *COL13A1*
**Postsynaptic**	
Endplate AChR deficiency	*CHRNA1*, *CHRNB1*, *CHRND*, *CHRNE*, *RAPSN*, *CHD8*, *MACF1*
CMS with arthrogryposis multiplex congenita	*CHRNA1*, *CHRND*, *CHRNG*, *MUSK*, *RAPSN*, *DOK7*, *SLC18A3*, *UNC50*
AChR anchoring, trafficking and stability	*RAPSN*, *CHD8*, *MACF1*, *UNC50*
SCCMS	*CHRND*, *CHRNE*
FCCMS	*CHRNA*, *CHRNB*
Sodium channel CMS	*SCN4A*
AChR-clustering defects	*AGRN*, *LRP4*, *MUSK*, *DOK7*
Glycosylation-deficient CMS	*GFPT1*, *DPAGT1*, *ALG2*, *ALG14*, *GMPPB*
Defects in postsynaptic cytoskeleton	*PLEC*, *DES*
Defects in nuclear structure	*TOR1AIP*
CMS associated with developmental disorders	*PURA*, *PTPN11*

This study aims to review the main aspects of CMS and explore aspects related to the LRP4 protein, demonstrating the physiology, systemic manifestations, and therapeutic features related to the deficiency of this protein. To identify the most relevant body of literature for the development of this narrative review, the authors selected PubMed (National Library of Medicine, National Center for Biotechnology Information) as the primary database. Articles published in all languages were initially considered. A preliminary search using the term “Congenital Myasthenic Syndrome” revealed 1295 records, including 406 case reports, 11 meta-analyses and systematic reviews, and 6 clinical trials.

To further refine the search strategy and increase specificity, two additional search approaches were employed. The first strategy, “(LRP4) AND (congenital myasthenic syndromes)”, yielded 50 results, including 24 review articles and 4 case reports, of which two were directly related to *LRP4*-associated CMS. No randomized controlled trials, clinical trials, meta-analyses, or systematic reviews were identified using this search strategy. The second strategy, “(Congenital Myasthenia) AND (LRP4)”, retrieved 55 records, including 5 case reports and 28 review articles. Similarly, no randomized controlled trials, clinical trials, meta-analyses, or systematic reviews were identified. Additionally, Google Scholar was used as a supplementary source for article selection. The search strategy “LRP4” AND “congenital myasthenic syndrome” enabled the identification of two additional case reports that were considered relevant to the present review.

## 2. Physiology of NMJ and LRP4 Protein

### 2.1. Organization and Physiology of the NMJ

The NMJ is a specialized structure of communication where signal transmission occurs between the nervous system and skeletal muscles. The NMJ is composed of three main components: presynaptic, synaptic, and postsynaptic. The presynaptic component corresponds to the motor nerve terminal, which arises from the axon of the α-motor neuron. The cell body of this neuron is located the anterior horn of the spinal cord or within the motor nuclei of the brainstem. This terminal is characterized by an abundance of synaptic vesicles containing acetylcholine (ACh), which is responsible for signal propagation. These vesicles are organized into distinct functional pools: vesicles in the active zones of the presynaptic membrane constitute the readily releasable pool of ACh, whereas reserve pools can be mobilized during intense synaptic activity. At the active zones, vesicle fusion and ACh release occur, precisely aligned with regions of high acetylcholine receptor (AChR) density on the postsynaptic membrane, thereby optimizing synaptic transmission. The differentiation and maintenance of the presynaptic terminal depend on retrograde signals originating from the muscle fiber, including molecules such as laminin β2, LRP4, and components of the Wnt signaling pathway. The synaptic cleft is 50–100 nanometers wide and separates the pre- and postsynaptic membranes, sharing an extracellular matrix composed of laminins, collagen Q, agrin, perlecan, biglycan, and acetylcholinesterase (AChE). This matrix provides structural support and contains molecules and enzymes that regulate synaptic transmission and NMJ homeostasis. Finally, the postsynaptic membrane exhibits junctional folds, which are membrane invaginations that markedly increase the surface area available for the insertion of receptors and ion channels. The crests of these folds contain a high density of AChRs, reaching approximately 10,000–20,000 receptors per μm^2^. This region is enriched with several structural and signaling proteins, including MuSK, LRP4, rapsyn, Dok-7, cytoskeletal proteins (actin, dystrophin, and components of the dystrophin–glycoprotein complex), and voltage-gated sodium channels [12,13].

### 2.2. LRP4 Protein

Low-density lipoprotein receptor-related protein 4 (LRP4), also known as Multiple Epidermal Growth Factor-like Domains 7 (MEGF7), is a member of the low-density lipoprotein receptor (LDLR) family and is encoded by the *LRP4* gene (11p11.2). The structure is composed of a short C-terminal region, a transmembrane domain, and a large extracellular N-terminal region with eight LDLa, followed by four homologous YWTD motif-containing β-propeller domains, which are interleaved by EGF-like modules [14]. At first, LRP4 was only known for its role in limb development and Wnt signaling [15,16]. However, it is now known that LRP4 plays an important role in the formation and maintenance of the NMJ. Genetic studies have shown that mice deficient in LRP4 exhibit pre- and postsynaptic differentiation defects similar to those observed in MuSK-mutant mice, as well as neonatal lethality [17].

### 2.3. Agrin–LRP4–MuSK Signaling Pathway

**Figure 1:**
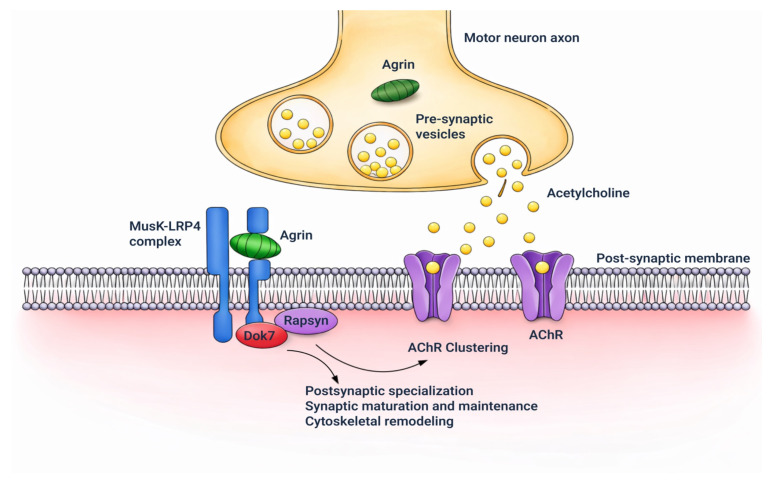
Schematic representation of the MuSK signaling pathway at the neuromuscular junction, illustrating the interaction between the presynaptic terminal (motor neuron) and the postsynaptic membrane (muscle fiber). The agrin protein, released by the nerve terminal, binds to the MuSK-LRP4 receptor complex on the muscle membrane. This binding activates the adaptor protein Dok7, which in turn recruits Rapsyn. Rapsyn mediates the clustering of acetylcholine receptors (AChR), concentrating them at the synaptic region to efficiently receive the acetylcholine (ACh) released from the vesicles. In addition, the activation of the MuSK-LRP4 complex triggers essential intracellular pathways for postsynaptic specialization, synaptic maturation and maintenance, and cytoskeletal remodeling.

Among the various molecular mechanisms underlying NMJ stability, the agrin–LRP4–MuSK signaling pathway represents a central axis that orchestrates postsynaptic differentiation and the clustering of acetylcholine receptors (AChRs) on the muscle membrane. This cascade is initiated by the secretion of agrin from motor neuron terminals, where it accumulates in the synaptic basal lamina and interacts with the receptor LRP4 expressed on the surface of muscle fibers. Agrin–LRP4 binding induces conformational changes that allow MuSK phosphorylation [18,19,20]. This is a key step in MuSK activation and downstream signaling (Figure 1), which was explored in the systematic study by Zong Y et al., which mapped the minimum agrin-binding domain of LRP4 to its first β-propeller domain [20]. The crystal structure of the complex formed between the LG3 domain of agrin and the β-propeller domain of the coreceptor LRP4 was determined, demonstrating the selectivity of agrin through a highly specific interaction mediated by an alternative splice insert exclusive to the neuronal isoform, termed z8. Structural analysis revealed that agrin functions as a monomeric ligand and that its ability to initiate signaling does not depend on intrinsic dimerization, but rather on the formation of a binary complex with LRP4. At the molecular level, this interaction is predominantly mediated by two residues located at the tip of the z8 insert, Asn1783 and Ile1785 [20]. Asn1783 establishes a network of hydrogen bonds with conserved residues of LRP4, whereas Ile1785 inserts into a deep hydrophobic pocket formed by apolar residues on the receptor surface [20]. Site-directed mutagenesis experiments, in which substitution of Asn1783 or Ile1785 abolished agrin binding to LRP4, impaired MuSK phosphorylation, and eliminated the ability to induce acetylcholine receptor clustering, without affecting the overall stability of the protein [20,21,22]. MuSK phosphorylation also requires Dok7 (*DOK7*), as studies in Dok7-deficient mice demonstrated that agrin failed to induce MuSK autophosphorylation or AChR clustering, which could be rescued by exogenous expression of Dok7 [22,23,24]. Subsequently phosphorylates AChR β1 subunit (*CHRNB1*), which binds to rapsyn (*RAPSN*) with a stoichiometry of 2:1 or 1:1 to form AChR clusters at the motor endplate [25]. Rapsyn anchors AChRs at the postsynaptic membrane by forming membraneless condensates through phase separation [25,26]. It also interacts with β-catenin (*CTNNB1*) and CHD8 (*CHD8*), reinforcing the complex, which is enhanced by Wnt [25,26,27,28]. LRP4 also mediates retrograde signaling from the postsynaptic membrane to the nerve terminal [29,30]. Furthermore, secreted molecules such as Rspo2, Fgf18, and Ctgf/Ccn2 at the NMJ enhance agrin–LRP4–MuSK signaling and promote NMJ formation [30,31,32,33].

## 3. Clinical, Electrophysiological, Laboratory, and Genetic Aspects of Congenital Myasthenic Syndromes

### 3.1. Clinical Features

Generally, CMS are characterized by fatigability, reduced muscle strength, and muscular hypoplasia, but some variants have distinct phenotypes [10]. Most CMS cases develop before the individual in question turns two years old; however, in some patients, symptoms start immediately after birth, or, rarely, at an even later age [34]. The symptoms typically improve throughout the years and may have spontaneous exacerbations or be triggered by febrile illness, stress, or intense activity. Exacerbation has been described in pregnant patients, but clinical features were, in general, transient [35]. Newborns with CMS regularly demonstrate ptosis, which is not expected in transient neonatal MG. AChR deficiency, AChE deficiency, and FCCMS subtypes are typically associated with ophthalmoplegia as well as bulbar and respiratory muscle weakness [36]. Nevertheless, a study of CMS-22 in China revealed feeding difficulties, profound hypotonia, and delayed motor development but only minor ocular findings [37]. Furthermore, arthrogryposis was found in some cases, especially at birth [38,39]. CMS has also been associated with life-threatening episodic apnea and reinforces the importance of recognizing CMS in several cases of SIDS [40,41].

### 3.2. Electrophysiological Examinations

Electrophysiological examination objectively demonstrates impaired neuromuscular transmission, which is important in the diagnosis and characterization of CMS. Repetitive nerve stimulation (RNS) at 2–3 Hz is the mainstay technique, revealing decrements of 10% or more of the compound muscle action potential (CMAP) amplitude, which are critical for the differential diagnosis of other neuromuscular disorders [42,43,44,45]. In performing RNS, it is crucial to ensure adequate placement of recording electrodes since muscle twitch induced by the first electrical stimulus moves the electrodes and changes the height and area of the CMAP, which could lead to a reading of false decremental response. Single-fiber electromyography (SFEMG) is the most sensitive method for detecting neuromuscular transmission failure by increased jitter and blocking, and is especially useful when RNS findings are subtle or negative (e.g., AChE deficiency) [43,44,45]. Despite having lower specificity and being technically challenging, many cases of CMS were diagnosed only by SFEMG [46]. A single nerve stimulus elicits a repetitive CMAP (R-CMAP) in some patients with *PURA*-CMS, SCCMS, and *COLQ*-CMS, and this finding rapidly disappears with RNS or spontaneous exercise, and a single nerve stimulus after a prolonged rest is important [47,48,49].

### 3.3. Muscle Biopsy and Creatine Kinase (CK)

Muscle biopsy and CK testing play important roles in the differential diagnosis of several neuromuscular disorders and may disclose findings specific to some CMS subtypes, although they are not primary diagnostic tools for CMS. Thus, these tests are most valuable as complements to electrophysiological and clinical findings that are inconclusive [43,49,50]. In most cases, a muscle biopsy demonstrates non-specific features such as mild variation in fiber size. Although *GFPT1*-CMS [51,52,53,54], *DPAGT1*-CMS [55,56,57], and *ALG2*-CMS [58] may show tubular aggregates or rimmed vacuoles associated with glycosylation defects.

Serum CK levels are typically normal. CK testing is primarily used to differentiate CMS from myopathies and muscular dystrophies, which typically show higher CK levels [59]. However, serum CK levels are above the upper limit of normal in endplate myopathies in SCCMS (~1.5 times), in patients with tubular aggregates in *GFPT1*-CMS and *DOK7*-CMS (~3 times), and in *GMPPB*-CMS (2 to 24 times) [60,61].

### 3.4. Pattern of Inheritance

The inheritance pattern of CMS is typically autosomal recessive. However, autosomal dominant inheritance or hemiallelic pathogenic variants are observed in five forms of CMS: SCCMS [3], *SNAP25*-CMS [62,63], *PURA*-CMS [64], *PTPN11*-CMS [65], and some patients with *SYT2*-CMS [66,67,68], but there are exceptions [69,70,71]. SCCMS is caused by a gain-of-function missense variant in a single allele that prolongs the channel openings of AChRs (when only 50% of AChRs at the NMJ are abnormal, SCCMS can develop) [3]. *SNAP25*-CMS [62,63] and *SYT2*-CMS [66,67,68,69,70,71] show LEMS-like CMS, probably due to dominant negative effects. *PURA*-CMS is believed to be caused by a hemiallelic loss of function of PURA [64]. *PTPN11*-CMS is associated with the gain-of-function of PTPN11 [65].

## 4. Systemic Manifestations Related to Variants of the LRP4 Gene

### 4.1. Congenital Myasthenic Syndrome Type 17 (CMS17; MIM #616304)

**Figure 2:**
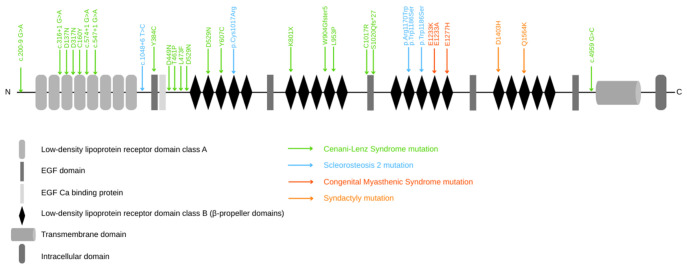
LRP4 protein structure, its domains, and the pathogenic variants related to the different *LRP4*-related diseases. Authorial source based on Masingue et al., 2023 [76].

LRP4 gene pathogenic variants have been associated with both inherited bone diseases and inherited NMJ disorders. Variants involving the third beta-propeller domain of LRP4 are associated with marked impairment of Wnt signaling, previously demonstrated in inherited bone diseases. On the other hand, variants at the edge of this domain have been associated with abnormal signaling with the MuSK protein and its associated complex with agrin, resulting in a new specific subtype of CMS, the so-called CMS type 17 [72,73,74,75]. It is noteworthy, however, that in the French cohort of individuals with CMS, a homozygous pathogenic variant c.1820A>G (p.Tyr607Cys) in the *LRP4* (NM_002334.4) gene involving the beta1 propeller domain of LRP4 protein was identified in a patient with CMS and severe renal and limb malformation (agenesis of the hands and feet), dysmorphic features commonly identified in the syndromic presentation of Cenani–Lenz syndactyly syndrome (discussed in detail in Section 4.2). This pathogenic variant led to an abnormal decrease in AChR aggregation in cultured myotube cells and, furthermore, binding of agrin and Wnt11 ligands to the mutated domain of LRP4 [76]. A summary of the main pathogenic variants described previously in the *LRP4* gene is presented in Figure 2.

CMS type 17 represents a rare genetic subtype of CMS with an autosomal recessive pattern of inheritance, which most commonly presents at birth or during infancy. Patients most typically present with global hypotonia, limb-girdle muscle weakness with reduced tendon reflexes, respiratory involvement, mild ocular involvement (mild ptosis, mild ophthalmoparesis), and variable bulbar compromise [72,73,74,75]. A summary of the currently described cases of CMS type 17 is presented in Table 2. Very early-onset phenotypes have been described previously. A first report included a 17-year-old woman who had been born at term and had presented with neonatal respiratory distress, including respiratory arrest and prolonged respiratory and feeding support until six months of age. She had significant fatigability during infancy, despite apparently normal motor developmental milestones and being at the upper limit of normal gait acquisition. During childhood, marked fatigability evolved, and she could not climb steps, thereby becoming partially wheelchair dependent. Neurological examination disclosed mild horizontal ophthalmoparesis, mild eyelid ptosis, moderately severe limb-girdle weakness, and reduced tendon reflexes. Repetitive nerve stimulation disclosed a 13–16% decremental response of the fourth compound muscle action potential of the spinal accessory nerve. There was no evidence of abnormal kinetics of the AChR channel. Genetic testing disclosed the presence of the compound heterozygous pathogenic variants c.3697G>A (p.Glu1233Lys) and c.3830G>A (p.Arg1277His) in the *LRP4* gene, affecting the third beta-propeller domain of the LRP4 protein. The patient had a previous worsening of motor symptoms after cholinesterase inhibitor use (pyridostigmine) [72].

Another report involving the homozygous pathogenic variant c.3698A>C (p.Glu1233Ala) at the edge of the third beta-propeller domain of LRP4 described a female neonate with a lethal and complex neonatal neuromuscular phenotype, disclosing severe global hypotonia, pulmonary hypertension, congenital diaphragmatic hernia, and progressive hypoxemia. This similar phenotype was also observed previously in her two siblings, who died in the newborn period [75].

Later, a 34-year-old woman and a 20-year-old Arab woman were also reported. The 34-year-old woman presented with a long-standing history of falls since infancy, generalized fatigue, mainly involving proximal muscle groups in the limbs (eventually becoming wheelchair-bound during adolescence), reduced tendon reflexes, hyperlordosis, waddling gait, severe involvement of the dorsal forearm muscles, and reduced vital capacity. Repetitive nerve stimulation disclosed a decremental response of 37% of the fourth compound muscle action potential in the trapezius and 14% in the facial muscle groups at 2 Hz. She had marked improvement of weakness and fatigability after albuterol therapy and worsening after pyridostigmine or 3,4-diaminopyridine. The 20-year-old woman, sister of the 34-year-old woman, had milder motor symptoms since infancy, mainly affecting the proximal muscle groups of the limbs, which markedly worsened at age 18 years, requiring assistance to climb a few steps and to rise from a sitting position. Examination disclosed reduced tendon reflexes, moderately severe involvement of the pelvic girdle muscles, and mild involvement of the cervical, shoulder, and arm muscle groups. There was also reduced vital capacity. There was a significant improvement in weakness after albuterol therapy and no changes after pyridostigmine. In both sisters, the homozygous pathogenic variant c.3698A>C (p.Glu1233Ala) in the *LRP4* gene was identified by whole-exome sequencing. This variant involves the central cavity of the third beta-propeller domain of the LRP4 protein [73]. In the adult group of individuals with CMS from the Mayo Clinic database followed in the neuromuscular clinic between 2000 and 2016, two sisters with *LRP4*-related CMS were described, and both had improvement with 3,4-diaminopyridine, with one showing improvement with albuterol [74].

The complex phenotype of CMS associated with abnormal skeletal and renal features was described in a 42-year-old Algerian woman, who presented with syndactyly of the hands and feet, severe brachydactyly of the hands, horseshoe-shaped kidney malformation, and early adult-onset of asymmetric proximal muscle weakness, fatigability, and cervical and abdominal weakness, which were transient at disease onset but remained permanent and progressive after three to four years from symptom onset. No significant bulbar involvement was identified. Eyelid ptosis and ophthalmoparesis were also not observed. Neurophysiological studies disclosed the occurrence of a 25–28% decremental response in the compound muscle action potentials of the spinal accessory nerves at 3 Hz repetitive nerve stimulation. Muscle biopsy disclosed nonspecific findings, including mild fiber size variability and marked type I fiber predominance. There was no significant improvement in CMS symptoms and signs after acetylcholinesterase inhibitor therapy [76].

In contrast to CMS17, anti-LRP4 myasthenia gravis (MG) is an acquired autoimmune disorder caused by IgG autoantibodies directed against LRP4. These antibodies can be detected in 1–5% of all patients with MG and 7–32,7% of double-seronegative patients (i.e., those negative for anti-AChR and anti-MuSK antibodies). Anti-LRP4 antibodies disrupt the agrin–LRP4 interaction, impairing AChR clustering and synaptic maintenance. Most patients who are exclusively LRP4-positive present with isolated ocular symptoms or with a milder form of generalized MG. While repetitive nerve stimulation may show a decremental response, it is more frequently normal in anti-LRP4 patients compared with other MG subtypes. Affected individuals usually respond well to AChE inhibitors and conventional immunotherapy [77,78,79].

### 4.2. Cenani-Lenz Syndactyly Syndrome (MIM #212780)

Cenani–Lenz syndactyly syndrome (CLSL) represents a rare autosomal recessive genetic disorder associated with homozygous or compound heterozygous variants in the *LRP4* gene. This dysmorphic syndrome is characterized by the association of several different limb anomalies and minor facial dysmorphism, such as a prominent forehead, hypertelorism, and micrognathia. Abnormal limb development is mainly present in the distal bones of the upper limbs, including abnormal morphology and fusion of the phalangeal and metacarpal bones, partial or total syndactyly of the hand, ulnar and radial shortening, radioulnar fusion, and even syndactyly of the feet. Systemic involvement is mainly observed as renal hypoplasia and, more rarely, kidney agenesis [80,81]. It is important to highlight that variants in the fourth beta-propeller domain of LRP4 have been associated with isolated syndactyly and fusion of the third and fourth fingers and not with a typical syndromic phenotype, such as that observed in CLSL and sclerosteosis type 2 (discussed in detail in Section 4.3) [82].

### 4.3. Sclerosteosis Type 2 (MIM #614305)

Sclerosteosis type 2 represents a rare, severe, and disabling autosomal dominant or recessive inherited disorder associated with homozygous, compound heterozygous, or heterozygous variants in the *LRP4* gene. This sclerosing bone dysplasia is associated with skeletal overgrowth, leading to different clinical complications, including facial dysmorphism (i.e., frontal bossing, hypertelorism, prognathism, macrocrania), sclerotic bone lesions (i.e., dense and enlarged clavicles, jaws, ribs, calvarium, and hips), hyperostosis of long bones, bony and cutaneous syndactyly of the fingers, increased stature, hearing loss, dysplastic nails, and motor complications due to direct compressive mechanism, such as peripheral facial nerve palsy and spastic-ataxic syndrome. Several facial dysmorphic features in sclerosteosis type 2 are shared with CLSL. Most cases have been previously described in the Afrikaner population in South Africa, while other cases have also been described in individuals of Greek and Spanish origin [82,83,84].

## 5. Therapeutic Care of Diseases Related to the LRP4 Gene

### 5.1. General Management of CMS

No licensed drugs are available specifically to treat any type of CMS, and all the drugs used are off-label. Given the heterogeneous molecular mechanisms involved, treatment differs greatly among distinct subtypes of CMS. Importantly, some drugs that benefit some subtypes may worsen symptoms in others, which illustrates the importance of genetic diagnosis. AChE inhibitors are generally first-line therapy in presynaptic CMS (e.g., ChAT deficiency, reduced synaptic vesicle release), AChR deficiency, fast-channel syndrome, sodium channel-related CMS (*SCN4A*), and glycosylation defect-related CMS (e.g., *GFPT1*). In contrast, they may worsen symptoms in acetylcholinesterase deficiency, slow-channel syndrome, and defects involving the agrin–LRP4–MuSK–Dok-7 pathway. Conversely, 3,4-DAP acts as a potassium channel blocker at the presynaptic terminal, thereby increasing ACh release. It may be beneficial in primary AChR deficiency and in selected postsynaptic subtypes, including fast-channel syndrome, MuSK deficiency, and rapsyn-related CMS. It has also been reported to be helpful in certain glycosylation defects (e.g., *ALG2*, *ALG14*, *GFPT1*, *DPAGT1*) and in rare presynaptic forms such as *SNAP25*-related CMS [11,43,85].

Beta-2-adrenergic receptor agonists, including salbutamol and albuterol, are thought to act by stabilizing the neuromuscular junction structure. These drugs are able to stimulate beta-2-adrenergic receptors, leading to activation and upregulation of cyclic AMP and protein kinase pathways, giving rise to improved stabilization of the NMJ, compensating for impaired synaptic signaling quality in individuals with some subtypes of CMS (like *DOK7*, *LRP4*, *MUSK*, *RAPSN*, and *AGRN*) and improving overall muscle activation, fatigue, and strength. These drugs are effective for several synaptic and postsynaptic CMS subtypes, particularly *DOK7*, *RAPSN*, primary AChR deficiency, and *COL13A1*-related CMS, and may also provide benefit in acetylcholinesterase deficiency. Ephedrine, an alpha- and beta-adrenergic receptor agonist, may also be effective in synaptic and post-synaptic forms, including laminin-β2 deficiency, agrin deficiency, acetylcholinesterase deficiency, and *DOK7*-related CMS [85]. Fluoxetine and quinidine act as open-channel blockers of muscle AChRs and are the first-line treatments for slow-channel CMS [85,86,87].

It is also highly recommended that patients with CMS avoid the routine use of drugs potentially harmful to the NMJ, similar to the guidelines and recommendations used in Myasthenia Gravis [85,88]. It is suggested that patients with CMS should avoid the use of drugs from therapeutic classes recommended to be avoided due to different harmful effects on the NMJ, such as nondepolarizing neuromuscular blockade agents, magnesium, aminoglycosides, macrolides, fluoroquinolones, class Ia antiarrhythmics (i.e., procainamide), beta-blockers (beta-adrenoceptor antagonists or blocking agents), and botulinum toxin [88]. In these scenarios, it is recommended to look for alternative therapeutic options for patients with any CMS subtype. It is important to highlight that cases of SCCMS are potentially treated with quinine, which differs from the differences in this context from other CMS subtypes [85]. There is, however, currently no evidence that other drugs, which change immune response patterns and which were associated with acute exacerbation, myasthenic crisis, or de novo Myasthenia Gravis, such as D-penicillamine, statins, and immune checkpoint inhibitors, have any type of impact on any genetic subtype of CMS [88,89].

### 5.2. Proposed Management of CMS Type 17

It is recommended that all suspected cases of CMS type 17 be confirmed by diagnostic testing to enable appropriate clinical management and therapeutic decisions, as with all other genetic subtypes of CMS [89]. Motor physiotherapy and respiratory support management approaches are also of great value, especially in early-onset and neonatal-onset cases of CMS type 17. It is important to use proper scores related to myasthenic syndromes during follow-up of adult and child patients with CMS, including the Myasthenia Gravis Activities of Daily Living (MG-ADL) score, the Quantitative Myasthenia Gravis (QMG) score, and the Myasthenia Gravis Composite Score (MGCS). It is also suggested to classify patients according to the Myasthenia Gravis Foundation of America (MGFA) class. Other patient-related outcome measures may also be used during follow-up [90].

Most therapeutic recommendations currently stem from case series, case reports, and reviews by neuromuscular specialists and geneticists, disclosing perspectives similar to those for other CMS subtypes related to the maintenance and development of the endplate and NMJ, especially *MUSK*-related CMS (or CMS type 9) [73]. There have been no specific clinical trials designed to treat CMS type 17. Most studies that previously evaluated the use of symptomatic therapies for CMS type 17 included mainly case reports, case series, expert opinion, and guideline recommendations, and results observed in preclinical studies in mouse models, thus indicating a very low level of evidence (recommendation grade C; level of evidence 4). No randomized controlled trials, meta-analyses, or systematic reviews have been conducted to evaluate therapeutic efficacy in the context of CMS type 17. Salbutamol and ephedrine have been consistently highlighted as the first-line therapies for CMS type 17 [91]. It is also important to consider the avoidance of pyridostigmine as it may worsen myasthenic symptoms, similarly to *MUSK*- and *DOK7*-related CMS, respectively, CMS types 9 and 10 [10,72,91]. Furthermore, albuterol sulfate has been previously associated with clinical and neurophysiological improvement due to its beta2 adrenergic receptor agonist effect, improving the ability of LRP4 to bind to MuSK, enabling its phosphorylation and activation [73], similarly to salbutamol and ephedrine. There is currently no evidence to support the use of 3,4-diaminopyridine, acetazolamide, fluoxetine, or quinidine in CMS type 17 [91], despite the common off-label use of 3,4-diaminopyridine, for example, in refractory cases of CMS. There have also been case descriptions reporting potential worsening of motor symptoms after the use of 3,4-diaminopyridine and pyridostigmine [73].

There is also no evidence that classical immunosuppressive therapies used to treat LRP4 antibody-associated Myasthenia Gravis have any clinical effect on the clinical management of CMS type 17. The same type of recommendations related to the avoidance of drugs potentially harmful to the NMJ should be used in CMS type 17, as previously discussed [88,89].

There are currently new therapeutic approaches under development for the treatment of CMS related to dysfunction of the development and maintenance of the endplate, such as *MUSK*-, *DOK7*-, and *LRP4*-related CMS [92]. It is believed, based on evidence from preclinical studies, that MuSK agonist molecules may enhance MuSK activation, thereby improving or stabilizing NMJ function [92]. ARGX-119 represents an intravenous humanized IgG1 monoclonal antibody agonist specific for MuSK, which has been shown to provide better functional and motor outcomes in preclinical mouse models of *MUSK*- and *DOK7*-related CMS, without significant interference with the binding of MuSK to neural agrin [92]. Another first proof-of-concept study using a passive transfer model induced by polyclonal patient IgG4 also reported improved survival and motor function in mice after ARGX-119 administration [93]. A Phase 1b randomized, double-blinded, placebo-controlled study (NCT06436742) was developed for patients with *DOK7*-CMS and, according to preliminary results reported by the sponsor, demonstrated favorable safety and tolerability profiles, besides significant improvements after 12 weeks in other secondary and exploratory endpoints, including the QMG score, the MG-ADL score, and the Six Minute Walk Test (6MWT) [94,95]. New studies with ARGX-119 in clinical trials for 5q Spinal Muscular Atrophy (Phase 2; NCT07287982) and Amyotrophic Lateral Sclerosis (Phase 2; NCT06441682) are currently being performed. It is currently possible to believe in the potential this therapy may show in future stages of clinical study development and in other forms of CMS, such as CMS type 17.

## 6. Conclusions

CMS represents a complex and heterogeneous group of inherited neuromuscular disorders, commonly underrecognized and misdiagnosed in clinical practice. The availability of specific therapeutic approaches and the occurrence of potentially serious neurological and systemic complications highlight the importance of early diagnosis and a high clinical suspicion. LRP4 protein dysfunction and *LRP4* pathogenic variants have been associated with other complex developmental and systemic phenotypes. *LRP4*-related CMS represents an autosomal recessive presentation associated with abnormal development and maintenance of the NMJ, which has been scarcely described in current literature. Low-cost symptomatic therapies are currently available to aid in the clinical management of adult and pediatric patients. New therapies are currently under development and have the potential to change the natural history of this rare inherited NMJ disorder.

## Figures and Tables

**Table 2:** Summary of the cases reported in the current literature involving CMS type 17 (*LRP4*-associated CMS). Variants are presented according to *LRP4* gene reference NM_002334.4. Legend: CMAP: Compound Muscle Action Potentials; EMG: electromyography; Ref.: Reference; RNS: repetitive nerve stimulation; y: years.

Ref.	Age at Diagnosis; Gender; Origin	Variant; Domain	Clinical Features	Laboratory Findings	Therapy Response
[72]	17 y; Female	c.3830G>A (p.Arg1277His), c.3697G>A (p.Glu1233Lys), compound heterozygous variants; third beta-propeller domain	Newborn respiratory distress, respiratory arrest, prolonged respiratory and feeding support up to 6 months of age; mild motor developmental delay; fatigability during infancy, progressing during childhood and adolescence; partially wheelchair dependent; mild ophthalmoparesis, mild eyelid ptosis, severe limb-girdle weakness	RNS with a 13–16% decremental response in the spinal accessory nerve. Muscle biopsy showed type I fiber preponderance.	Worsening of motor symptoms with pyridostigmine. Transient and partial improvement of decremental response after edrophonium chloride.
[75]	Newborn; Female	c.3698A>C (p.Glu1233Ala), homozygous; third beta-propeller domain	Lethal and complex neonatal neuromuscular phenotype: reduced fetal movements, severe global hypotonia, pulmonary hypertension, right-sided congenital diaphragmatic hernia, progressive hypoxemia, refractory hypotension; similar clinical phenotype seen in her two siblings (lethality during newborn period)	Neurophysiological testing and muscle biopsy were not performed	Data unavailable. Very early lethal clinical course.
[74]	Two sisters (patients from the Mayo Clinic database)—no additional epidemiological data available	Gene variants unavailable	Adult CMS cases with limb-girdle phenotype and the classic CMS phenotype	Both sisters had raised serum CK levels. One with unspecific muscle biopsy findings. One sister presented with fibrillation potentials on needle EMG studies.	One sister had improvement with albuterol. Both sisters had improvement with 3,4-diaminopyridine
[73]	34 y; Female; Arab	c.3698A>C (p.Glu1233Ala), homozygous; third beta-propeller domain	Falls since infancy, long-standing generalized fatigue, proximal-dominant quadriparesis, severe weakness of the dorsal forearm, hyperlordosis, waddling gait, wheelchair-bound during adolescence, reduced vital capacity (49%)	RNS with 37% of decremental response in the trapezius and 14% in the facial muscle groups at 2 Hz. Needle EMG with short-duration, polyphasic motor unit potentials without spontaneous electrical activity.	Marked improvement with albuterol (4 mg, twice daily) in clinical and neurophysiological parameters. Worsening with pyridostigmine and 3,4-diaminopyridine
[73]	20 y; Female; Arab	c.3698A>C (p.Glu1233Ala), homozygous; third beta-propeller domain	Very early onset of limb-girdle muscle weakness, mainly severe compromise of hip girdle muscles; hyperlordosis, waddling gait; reduced vital capacity (68%)	Needle EMG showing mild proximal myopathic involvement.	Improvement after albuterol (4 mg, twice daily). No changes with pyridostigmine.
[76]	42 y; Female; Algeria	c.1820A>G (p.Tyr607Cys), homozygous; beta1 propeller domain	Syndactyly of hands and feet; severe brachydactyly of the hands; horseshoe-shaped kidney malformation; early adulthood-onset of asymmetric proximal quadriparesis, fatigability, cervical and abdominal weakness; no eyelid ptosis, ophthalmoparesis, or bulbar involvement noted	RNS with a 25–28% decremental response in the CMAP of the spinal accessory nerve at 3 Hz. Muscle biopsy with marked type I fiber predominance and fiber size variability	No improvement with acetylcholinesterase inhibitor. Salbutamol not tested.

## Data Availability

Data sharing is not applicable (No original data were created).

## Abbreviations

6MWT	Six Minute Walk Test
ACh	Acetylcholine
AChE	Acetylcholinesterase
AChR	Acetylcholine Receptor
CLSL	Cenani-Lenz Syndactyly Syndrome
CMS	Congenital Myasthenic Syndrome
Dok7	Downstream-of-kinase or docking-protein 7
LDLR	Low-density lipoprotein receptor
LRP4	Low-density lipoprotein receptor-related protein 4
MEGF7	Multiple Epidermal Growth Factor-like Domains 7
MG-ADL	Myasthenia Gravis Activities of Daily Living
MGCS	Myasthenia Gravis Composite Score
NMJ	Neuromuscular Junction
QMG	Quantitative Myasthenia Gravis
SCCMS	Slow-channel Congenital Myasthenic Syndrome
